# Evaluation of the force generated by gradual deflection of 0.016-inch NiTi and stainless steel orthodontic wires in self-ligating metallic and esthetic brackets

**DOI:** 10.4317/jced.55698

**Published:** 2019-05-01

**Authors:** Manoela-Fávaro Francisconi, Guilherme Janson, José-Fernando-Castanha Henriques, Karina-Maria-Salvatore Freitas, Paulo-Afonso-Silveira Francisconi

**Affiliations:** 1 D.D.S., M.Sc. Orthodontic Graduate Student. Department of Orthodontics. Bauru Dental School. University of São Paulo, Bauru, Brazil; 2 D.D.S., M.Sc., Ph.D. Professor and Head. Department of Orthodontics. Bauru Dental School, University of São Paulo, Bauru, Brazil; 3 D.D.S., M.Sc., Ph.D. Professor. Department of Orthodontics. Bauru Dental School, University of São Paulo, Bauru, Brazil; 4 D.D.S., M.Sc. PhD. Professor. Department of Orthodontics. UNINGA University Center, Maringa, Brazil; 5 D.D.S., M.Sc., Ph.D. Associate Professor. Department of Operative Dentistry, Endodontics and Dental Materials. Bauru Dental School, University of São Paulo, Bauru, Brazil

## Abstract

**Background:**

The purpose of this study was to evaluate the deflection forces of 0.016-inch Nitinol and stainless steel orthodontic wires, in association to different self-ligating brackets.

**Material and Methods:**

Specimens were mounted in a clinical simulation model and evaluated in a Universal Testing Machine (INSTRON 3342), using a 10N load cell and ISO 15,841, as a protocol. Eight of these models were prepared, each one for the bonding of each set of self-ligating accessories to be tested: Damon Q, Damon Clear (Ormco), In-Ovation R, In-Ovation C (GAC), BioQuick, QuickClear (Forestadent), SmartClip and Clarity SL (3M). Data were subjected to One-way ANOVA, followed by Tukey tests (*P*<0.05).

**Results:**

Elastic deflection results showed that the deactivation forces increased with increase in wire deflection in the different brackets evaluated. For the different combinations, Clarity SL generated the greatest force and Damon Clear presented the lowest force when compared to the other brackets in all alloys and deflections. BioQuick and QuicKlear were those with the most similar behavior with each other.

**Conclusions:**

Strength values increased with gradual increase in wire deflection in all evaluated brackets. Clarity SL generated the greatest and Damon Clear the lowest force when compared to the other brackets in all alloys and deflections tested.

** Key words:**Brackets, orthodontic wires, deflection.

## Introduction

Nowadays, having a natural and pleasant smile even during orthodontic treatment is one of patients’ main concerns. Devices combining acceptable esthetic and adequate technical performance, satisfying both the patient and the clinician expectations, have been developed (1). Nevertheless, esthetic brackets show higher friction coefficients than metallic brackets, which can impair the desired movement (2).

Self-ligating brackets, introduced as Russel’s accessories in the mid-1930s are systems which present a mechanical device designed to close the edgewise slot (3). For patients, such brackets are usually more comfortable and easier to clean since elastic ligatures are not necessary (4). Reduced treatment time, seemingly related to a significant lower friction than that observed for conventional brackets, is another positive aspect of self-ligating brackets (5).

Therefore, effectiveness of orthodontic movement results not only from the different brackets systems, but also from a series of other factors, related to both the patient (teeth and supporting structures) and the type of mechanics applied. Teeth move also in dependence on the action of orthodontic wires, which varies according to their structural and mechanical properties (6).

Consequently, it is necessary to assess not only the friction related to different bracket systems but also the behavior of different currently available orthodontic materials regarding the forces applied during orthodontic mechanics. Furthermore, development of esthetic brackets with metal components brings up a new field for research (7). To make the best choice among the various brackets and orthodontic wires available, it is essential to know the magnitude of forces released by these wires and their behavior in relation to the gradual increase of wire deflection (8).

This in vitro study assessed deflection forces of 0.016-inch caliber Nitinol and stainless steel orthodontic wires, placed in self-ligating brackets, by using a clinical simulation model and following ISO 15,841 as protocol.

## Material and Methods

-Material - Experimental Groups

The sample used in this study consisted of 320 round-section, 0.016-inch diameter, Nitinol and stainless steel orthodontic wires (Standard or Medium, GAC®, Bohemia, NY, USA) and eight different self-ligating bracket types: Damon Q, Damon Clear (Ormco), In-Ovation R, In-Ovation C (GAC), BioQuick, QuickClear (Forestadent), SmartClip and ClaritySL (3M) ( Table 1).

-Methods - Clinical Simulation Device 

**Table 1:**
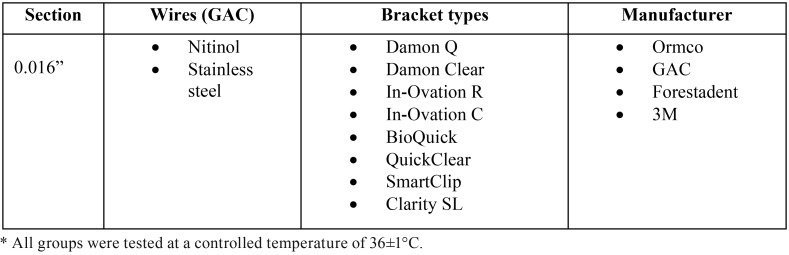
Sample used and test conditions*.

In order to internationally standardize the tests as adequately as possible, the methods used in this study followed the ISO 15,841 (International Organization for Standardization. ISO 15841: Dentistry - Wires for use in orthodontics. Berlin, 2006).

**Figure 1:**
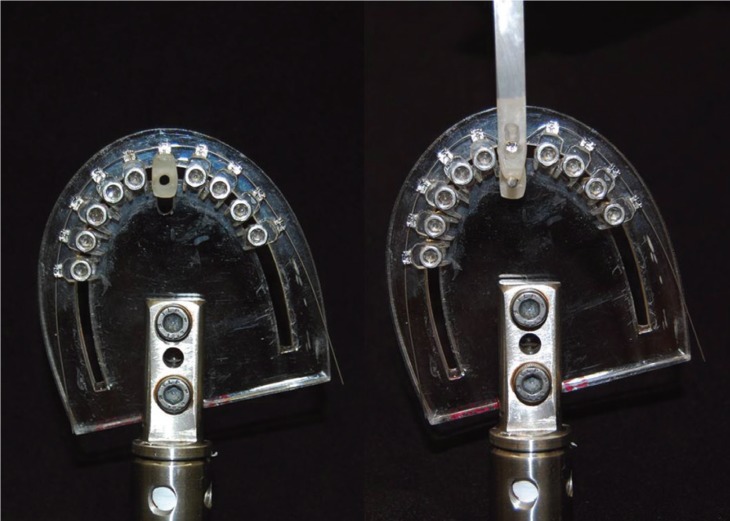
Clinical simulation device.

Deflection of the orthodontic wire was performed in a clinical simulation device representing all 10 teeth of the maxillary arch (9). Figure 1 shows the clinical simulation device that was used in this study. Brackets were bonded with cyanoacrylate ester gel (Super Bonder, Loctite) on the acrylic structures. These structures were fixed by means of threaded screw in the bottom of the acrylic resin plate.

The tests were performed on the structure corresponding to the right maxillary central incisor (Fig. 1). Unlike the others, this structure was not screwed, enabling its labio-lingual movement. It had a perforation, in which a metal cylinder was placed to activate it. The tip of the activation head, attached to the testing machine, had a rounded cut to fit the metal cylinder. Deflection of the wire was performed without changing the inter bracket distance (6mm), since the relation deflection/force is dependent, among other things, on this distance. The speed of the deflection was 2mm/min.

**Figure 2:**
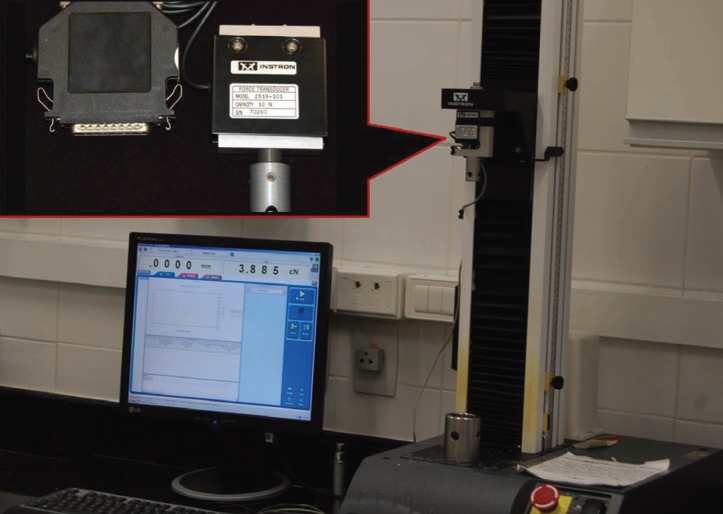
Instron universal testing machine used in this study, with a load cell of 10N.

**Figure 3:**
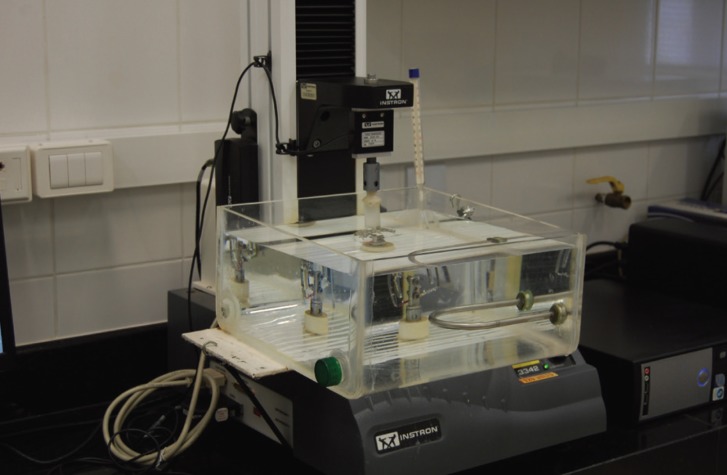
Acrylic container adapted to the Instron device.

Records of the force released by the wire deflection were made in 0.5, 1, 2 and 3mm. The deflection tests were performed using the Universal Testing Machine (Instron 3342), with a load cell of 10N (10)(Fig. 2). This load cell has an accuracy of 0.5% of the reading value with the temperature of 25° Celsius. In this study, the load cell was maintained at this temperature. Also according to the ISO standard, the tests were always performed at the same testing temperature of 36 ± 1° C for all test groups. To obtain this, an acrylic container with water at a temperature of 36 ± 1° C, maintained with the aid of submersible heater with integral thermostat (Electronic Atman Heater, China) and checked by a decimal precision thermometer, with a limit of error of ± 0.2° C (Incoterm, reference 5097, São Paulo, SP, Brazil), was adapted to the testing machine (11) (Fig. 3).

-Statistical Analyses

The sample size, according to the ISO 15,841 standards is of 6 specimens in each group. To minimize the chances of any technical error and increase reliability of the results, 20 specimens were chosen for each group. Outliers were excluded through a statistical program that provides the values to be deleted. Normal distribution was evaluated with Kolmogorov-Smirnov tests. Because all variables showed normal distribution, the following tests were used:

Comparison among different self-ligating brackets in NiTi and stainless steel 0.016-inch wires were performed with one-way ANOVA and Tukey tests.

All statistical analyses were performed with Statistica software (Statistica for Windows – Release 7.0 - Copyright Statsoft, Inc. Tulsa, Okla). Results were considered significant at *P*<0.05.

## Results

-Results of different self-ligating bracket types and nickel-titanium orthodontic wires

In general, it was observed that the deactivation force increased with the increase in amount of deflection ( Table 2). There was significant and progressive force increase with all amounts of deflections.

**Table 2:**
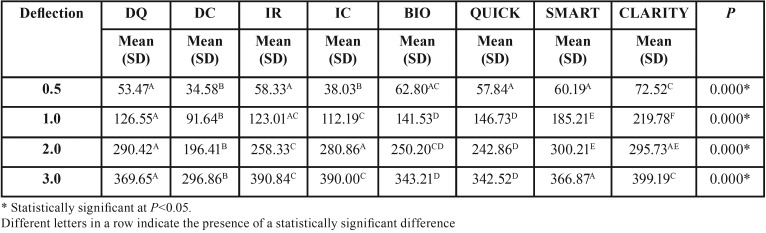
Deactivation forces (cN) comparisons of the self-ligating bracket types with 0.016-inch nickel-titanium wire, in progressive deflections (One-way Anova followed by Tukey tests).

However, with 0.016-inch Nitinol wires in 0.5mm of deflection, the results were not standardized between different self-ligating bracket combinations.

The deactivation forces were significantly higher in deflections of 1, 2 and 3mm with 0.016-inch nickel-titanium wires with self-ligating brackets ( Table 2). Overall, Clarity SL showed the highest while Damon Clear showed the lowest deactivation forces ( Table 2).

The results of combinations of different self-ligating bracket types with 0.016” Nitinol wires demonstrated that BioQuick and QuicKlear were those with the most similar behavior with each other ( Table 2).

-Results of different self-ligating bracket types and stainless steel orthodontic wires 

Again, it was observed that the deactivation force increased with the increase in amount of deflection ( Table 3).

**Table 3:**
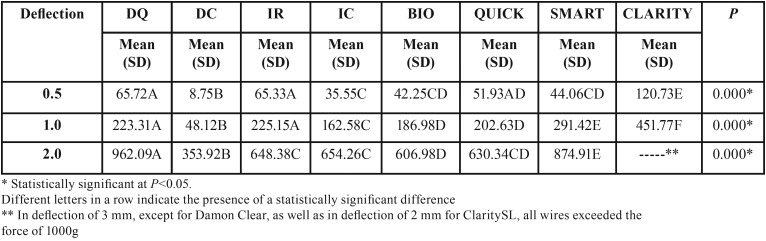
Deactivation forces (cN) comparisons of the self-ligating bracket types with 0.016-inch stainless steel wire, in progressive deflections (One-way Anova followed by Tukey tests).

The results of combinations of different self-ligating bracket types with 0.016” stainless steel wires demonstrated, once again, that BioQuick and QuicKlear were those with the most similar behavior with each other ( Table 3).

Finally, in deflection of 3mm, except for Damon Clear, as well as in deflection of 2mm for Clarity SL, all wires exceeded the force of 1000g. Once more, Clarity SL showed the highest while Damon Clear showed the lowest deactivation forces.

## Discussion

-Sample and methodology

A clinical simulation device was used to approximate the laboratory results to clinical situations, providing more practical applications (12). Even with this in mind, the specific ISO standard was used for orthodontic wires laboratory tests.

The elastic deflection test was chosen because it is clinically closest to the orthodontist’s interests, because that is what he does when adapting a wire to the patient’s teeth.

Nickel titanium and stainless steel wires were used because the authors were interested in examining unloading magnitude in the initial phase of orthodontic treatment when archwires with a low modulus of elasticity are indicated. Horizontal deflections of 0.016-inch wires were standardized at 3mm, following ISO 15,841 as protocol, with the purpose of enabling them to reach full superelastic property in the NiTi archwire, since these wires deflections around 2mm may be insufficient to bring out the superelastic properties of archwires (13).

-Results of comparisons between different combinations with nickel-titanium orthodontic wires

The results found in different combinations of the self-ligating brackets with 0.016-inch nickel-titanium wires are in agreement with other authors that have found that there was significant and progressive force increase with all amounts of deflection (14). This may be consequent to the more uniform wire mechanical locking system than the wire tying process of conventional brackets, with elastomeric ligatures ( Table 2) (15).

Although in 0.5mm of deflection, the results were not standardized between different self-ligating bracket combinations, another study found that designs of the brackets, which limited the wire tying strength, generate less friction at low deflections ( Table 2)(17).

Confirming previous results (17), there were no differences in the unloading forces between Damon Q and In-Ovation R self-ligating brackets with 0.016-inch NiTi wires in smaller deflections of 0.5 and 1.0mm ( Table 2).

Contrary to other study (18) that found no clinically relevant differences when comparing the deactivation forces produced by superelastic NiTi wires in different self-ligating brackets, significant differences among them were observed in this study ( Table 2).

The results of combinations of different self-ligating bracket types with 0.016-inch Nitinol wires in most deflections, demonstrated that passive self-ligating brackets, Damon Q, SmartClip and ClaritySL, generated significantly greater forces when compared to actives such as In-Ovation R, In-Ovation C, BioQuick and QuicKlear ( Table 2). Other authors (19) also noticed higher forces when passive (SmartClip) were compared to active self-ligating brackets (Time3). This result can be explained by assuming that part of the force is used to overcome the greater resistance to sliding, generated in tests with active self-ligating bracket systems during unloading (4). This result is consistent with another study in which friction was responsible for reducing the amount of released force (15). This partially explains the fact that the highest average force was generated by the simulation device with passive self-ligating brackets (20).

Clinically, this explanation makes sense because friction increases the released force during loading, but decreases it during unloading (13). Therefore, the device with higher friction generated less force, because during the deactivation, friction hinders the return of the wire to its initial position (15). The presence of the brackets, the distance between them, the bands and the crowding itself are factors that increase friction in the clinical setting. Thus, this large friction would be able to decrease the released force by the wire.

Damon Clear presented the smallest forces in all deflections when compared to the others, contradicting previous investigation ( Table 2)(19). But these results are in agreement with another investigation (20) which did not observe a consistent pattern among the tested brackets, even among the active and passive self-ligating, when the dissipated forces during a simulation of canine retraction, using different types of brackets, were compared.

On the other hand, Clarity SL showed the highest forces in all deflections when compared to the others ( Table 2). Clarity SL, classified as a passive self-ligating bracket, showed high deactivation forces. The reason may be its structure because Clarity SL is different than other passive self-ligating bracket designs. The Clarity SL structure design consists of two clips at the mesial and distal wings to hold the archwire. This arrangement also facilitates free movement of the archwire inside the bracket (21).

-Results of comparisons between different combinations with stainless steel orthodontic wires

The results found in different combinations of the self-ligating brackets with 0.016-inch stainless steel wires are in agreement with other authors (23) that have found that there was significant and progressive force increase with all amounts of deflection ( Table 3).

The results of combinations of different self-ligating bracket types with 0.016-inch stainless steel wires, in most deflections, are in agreement with other authors (23) that have demonstrated that polycrystalline self-ligating brackets with metal slots, Clarity SL, and stainless steel self-ligating brackets, SmartClip, generated significantly greater forces when compared to polycrystalline self-ligating brackets with a glazed slot, In-Ovation C ( Table 3). Ceramic brackets have shown consistently higher frictional resistance during sliding than stainless steel brackets or ceramic brackets with stainless steel slots hence, decreasing the deactivation forces (24).

Confirming previous results (25), there were no differences in the unloading forces between Damon Q and In-Ovation R self-ligating brackets with 0.016-inch stainless steel wires at low deflections of 0.5 and 1.0mm ( Table 3). Although Damon Q bracket has a ‘passive’ cap as opposed to an ‘active’ clip mechanism to retain the archwire, In-Ovation R has active clips that deliver an active force that forces the archwire into the slot, but only after the archwire exceeds a certain buccolingual (25).

Clarity SL showed the highest and Damon Clear showed the lowest deactivation forces (T Table 3). Another study (26) also noticed the maximum amount of kinetic forces with all types of wire dimensions and properties with Clarity SL, when compared to Damon Clear.

Thus, it was noticed that in sliding mechanics, the force applied to a tooth is not fully delivered to the periodontium because the friction force at the archwire/bracket interface opposes the sliding archwire and thereby dissipates part of the force designed to move teeth (5,27). Clinically, as justified earlier, this explanation makes sense because friction increases the released force during loading, but decreases it during unloading (13). Therefore, the device with higher friction generated less force, because during the deactivation, friction hinders the return of the wire to its initial position (15). Thus, the orthodontic forces must first overcome friction while the remaining force promotes bone remodeling, causing teeth movement (27).

Perhaps another important aspect is the fact that the different results are likely to be caused by the particular design of the Clarity SL bracket. With this bracket, the wire is tied by two NiTi clips and pressed into the slot so that a certain amount of pressure is exerted. In contrast, the locking cap in esthetic self-ligating brackets just passively converts the bracket slot into a tube, and hence, no pressure is exerted on the wire (26).

Other authors (28) also noticed higher frictional forces when active (Quick; Forestadent) were compared to passive self-ligating brackets (Damon3 MX; Ormco), with larger wires (23). Kusy (29) also reported that the passive self-ligating brackets exhibited low frictional forces and that the active self-ligating brackets showed varying degrees of frictional force. Ceramic brackets exhibit higher frictional forces than metal brackets, because the orthodontic wires bind more easily with ceramic brackets, which have rough surfaces as opposed to metal brackets, which have relatively polished and softer surfaces (24,29). Although ceramic brackets are esthetically pleasing, higher frictional forces inside the bracket slots than those in metal brackets are considered a disadvantage (30). According to Cacciafesta (24), variables that can affect the frictional force include orthodontic wires, brackets, ligation method, and orthodontic appliances, among others. Orthodontic wires vary in size, shape, and material, but overall, stainless steel wires cause the least frictional force.

Finally, in deflection of 3mm, except for Damon Clear, as well as in deflection of 2mm for Clarity SL, all wires exceeded the force of 1000g ( Table 3). Therefore, the deactivation forces were generally significantly higher with stainless steel wires than with Nitinol wires ( Table 2 and  Table 3). Other authors (21) concluded that the influence of archwire alloys on the frictional properties of various self-ligating mechanisms was highly significant.

## Conclusions

• The deactivation forces increased with increase in wire deflection in the different brackets evaluated;

• Clarity SL generated the greatest and Damon Clear the lowest force when compared to the other brackets in all alloys and deflections tested.
